# A combined epigenome- and transcriptome-wide association study of the oral masticatory mucosa assigns *CYP1B1* a central role for epithelial health in smokers

**DOI:** 10.1186/s13148-019-0697-y

**Published:** 2019-07-22

**Authors:** Gesa M. Richter, Jochen Kruppa, Matthias Munz, Ricarda Wiehe, Robert Häsler, Andre Franke, Orlando Martins, Yvonne Jockel-Schneider, Corinna Bruckmann, Henrik Dommisch, Arne S. Schaefer

**Affiliations:** 1 Department of Periodontology and Synoptic Dentistry, Institute for Dental and Craniofacial Sciences, Charité – Universitätsmedizin Berlin, Freie Universität Berlin, Humboldt-Universität zu Berlin, and Berlin Institute of Health, Aßmannshauser Str. 4-6, 14197 Berlin, Germany; 2 Institute for Biometry and Clinical Epidemiology, Charité – Universitätsmedizin Berlin, Freie Universität Berlin, Humboldt-Universität zu Berlin, and Berlin Institute of Health, Charitéplatz 1, 10117 Berlin, Germany; 3 Medical Systems Biology Group, Institute of Experimental Dermatology, Institute for Cardiogenetics, University of Lübeck, 23562 Lübeck, Germany; 4 Institute of Clinical Molecular Biology, Christian-Albrechts-University, Rosalind-Franklin-Straße 12, 24105 Kiel, Germany; 5 Institute of Periodontology, Dentistry Department, Faculty of Medicine, University of Coimbra, Av. Bissaya Barreto, Bloco de Celas, 3000-075 Coimbra, Portugal; 6 Department of Periodontology, Clinic of Preventive Dentistry and Periodontology, University Medical Center of the Julius-Maximilians-University, Pleicherwall, 97070 Würzburg, Germany; 7 Department of Conservative Dentistry and Periodontology, Medical University Vienna, School of Dentistry, Sensengasse 2a, 1090 Vienna, Austria

**Keywords:** EWAS, Methylation, Expression, Masticatory mucosa, *CYP1B1*, *AHRR*, Cytochrome P 450 pathway, OSCC, Smoking

## Abstract

**Background:**

The oral mucosa has an important role in maintaining barrier integrity at the gateway to the gastrointestinal and respiratory tracts. Smoking is a strong environmental risk factor for the common oral inflammatory disease periodontitis and oral cancer. Cigarette smoke affects gene methylation and expression in various tissues. This is the first epigenome-wide association study (EWAS) that aimed to identify biologically active methylation marks of the oral masticatory mucosa that are associated with smoking.

**Results:**

Ex vivo biopsies of 18 current smokers and 21 never smokers were analysed with the Infinium Methylation EPICBeadChip and combined with whole transcriptome RNA sequencing (RNA-Seq; 16 mio reads per sample) of the same samples. We analysed the associations of CpG methylation values with cigarette smoking and smoke pack year (SPY) levels in an analysis of covariance (ANCOVA). Nine CpGs were significantly associated with smoking status, with three CpGs mapping to the genetic region of *CYP1B1* (cytochrome P450 family 1 subfamily B member 1; best *p* = 5.5 × 10^−8^) and two mapping to *AHRR* (aryl-hydrocarbon receptor repressor; best *p* = 5.9 × 10^−9^). In the SPY analysis, 61 CpG sites at 52 loci showed significant associations of the quantity of smoking with changes in methylation values. Here, the most significant association located to the gene *CYP1B1*, with *p* = 4.0 × 10^−10^. RNA-Seq data showed significantly increased expression of *CYP1B1* in smokers compared to non-smokers (*p* = 2.2 × 10^−14^), together with 13 significantly upregulated transcripts. Six transcripts were significantly downregulated. No differential expression was observed for *AHRR*. In vitro studies with gingival fibroblasts showed that cigarette smoke extract directly upregulated the expression of *CYP1B1.*

**Conclusion:**

This study validated the established role of *CYP1B1* and *AHRR* in xenobiotic metabolism of tobacco smoke and highlights the importance of epigenetic regulation for these genes. For the first time, we give evidence of this role for the oral masticatory mucosa.

**Electronic supplementary material:**

The online version of this article (10.1186/s13148-019-0697-y) contains supplementary material, which is available to authorized users.

## Background

Smoking is considered one of the most important environmental factors causing premature death by promoting a variety of malignant and non-malignant diseases. It increases the relative risk of dying by up to 2.8 and shortens lifetime expectancies by 10 years on average [1]. The oral mucosa is the barrier tissue that is most directly exposed to cigarette smoke. Accordingly, smoking is a strong risk factor for a variety of oral diseases such as oral cancer [2] and the widespread common oral inflammatory disease periodontitis (PD) [3]. The deleterious effects of smoking on tissue integrity are driven by a variety of biological mechanisms. In the recent past, evidence grew that some of these mechanisms correlate with changes in DNA methylation patterns. DNA methylation plays a particular role in cell differentiation and the maintenance of cell specificity [4] and represents an important mechanism for cells to react on persistent external stimuli, allowing somatic cells to adjust gene expression to a particular environment in a long-term manner that can be passed on to daughter cells [5, 6]. Smoking is currently the best studied environmental factor altering DNA-methylation patterns [7]. A plethora of studies exist that show significantly different methylation patterns in smokers compared to non-smokers in different tissues [2, 8–22] and in the context of smoke-related diseases like cancer [23] or chronic obstructive pulmonary disease (COPD) [21]. The best-replicated gene-specific association of differential methylation with smoking is attributed to the aryl-hydrocarbon receptor repressor *AHRR* across various tissues and cell types [8, 10, 12–14, 16, 17, 20, 22, 24, 25]. *AHRR* is a core regulator in the aryl hydrocarbon receptor (AhR) signalling cascade, modulating dioxin toxicity pathways involving Cytochrome P450, most likely in a cell-specific manner [26]. Most EWAS that assessed the role of CpG methylation in tobacco-related xenobiotic metabolism analysed leukocytes, but some also analysed airway epithelial cells [27] and buccal cells [12, 21, 22]. These cell types are located at the interface to the environment and form a mechanical and physiological barrier. Most notably, a large comprehensive EWAS analysed buccal cells of 790 healthy individuals and identified a set of 1501 CpGs that were differentially methylated in smokers [22]. Buccal swabs largely consist of non-keratinized squamous epithelial cells detached from the inner facial cheeks, and immune cells from the saliva. In the current study, we chose to analyse the effects of cigarette smoke on the healthy keratinized oral mucosa, which is restricted to the gingiva, the tongue and the hard palate. It is the tissue that forms the mechanical barrier between the oral cavity and the hard tissues (alveolar bone and skull). It consists of an upper layer of highly keratinized stratified squamous epithelium, the underlying keratinized connective tissue and invaded immune cells, which make it a strong mechanical and immunological barrier against injuries and pathogens. In addition, the gingiva is the only interface of the human body that is penetrated by hard tissues (the teeth), forming a direct connection between the environment and the bone. In this context, the masticatory mucosa is unique. Accordingly, the gingiva is the tissue which is affected by the common inflammatory disease periodontitis, for which smoking is the strongest risk factor. In contrast, buccal cells are not involved in the pathophysiology of periodontitis. The different functions of the lining mucosa of the inner cheeks, where buccal cells are derived, and of the masticatory mucosa are characterised by pronounced differences in cell turnover [28], cellular differentiation [29], and differences in expression patterns and permeability [29, 30]. Because of these special features in cell derivation, function and clinical significance, the objective of our study was to assess the putative differences of methylation and transcription of the oral masticatory mucosa between healthy smokers and never smokers. Knowledge of these differences will provide information on the regulatory responses of this interface to direct exposure to xenobiotics of tobacco smoke before periodontitis or oral cancer becomes a clinical manifestation, and if defective, may have a relevant role in the disease aetiology.

To this end, we performed an EWAS with 39 healthy participants, all of them showing no signs of oral inflammation as indicated by a periodontal screening index (PSI) < 2. Taking into account that the masticatory mucosa itself also differs in cell type composition across different oral regions [31], samples were extracted from a clearly defined site. In this very homogeneous set of ex vivo biopsies, methylation patterns were investigated using the Infinium Methylation EPICBeadChip. In order to investigate potential effects of differentially methylated CpG sites on gene expression, we additionally performed RNA-sequencing (RNA-Seq) in the same samples. With this study, we provide a genome-wide methylation reference set and a RNA-Sequencing data set for the healthy masticatory mucosa, the clinically affected tissue in periodontitis. For the first time, smoking-informed reference datasets for methylation and expression patterns are combined for the oral mucosa. With these datasets, we were able to validate associations of CpGs with smoking levels shown in buccal swabs [22] and add to the knowledge of potential tissue-specific differential methylation in smokers. We showed shifts in transcriptional patterns of smokers, which partly paralleled the differential methylation patterns.

## Results

### DNA methylation differences of the masticatory mucosa between smokers and non-smokers

**Fig. 1:**
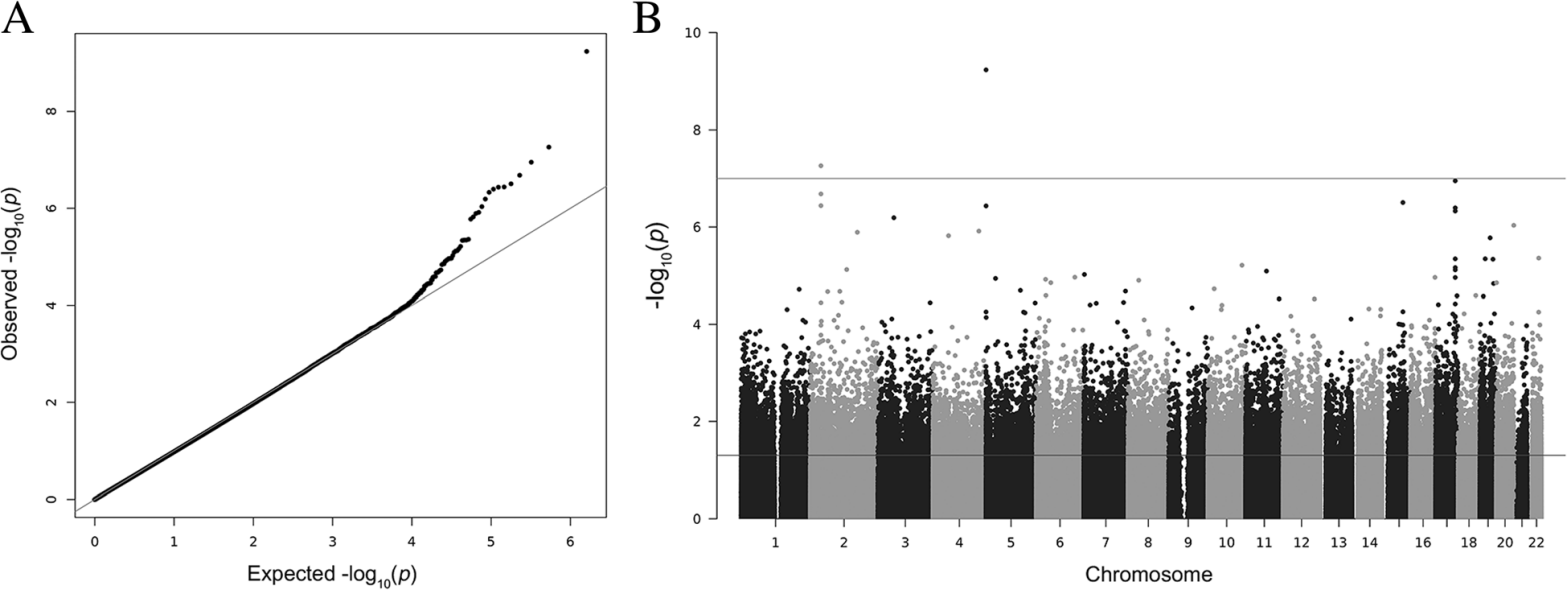
Manhattan and QQ plots for epigenome-wide associations with smoking status in the masticatory mucosa. **a** The QQ plot showed some evidence of inflated association signals (λ = 1.12). **b** Manhattan plot showing -log_10_ transformed *p* values from the ANCOVA plotted against the genomic location of the probes. The horizontal lines indicate the nominal significance threshold (*p* < 0.05) and the genome-wide significance threshold (*p* < 10^-7^)

**Fig. 2:**
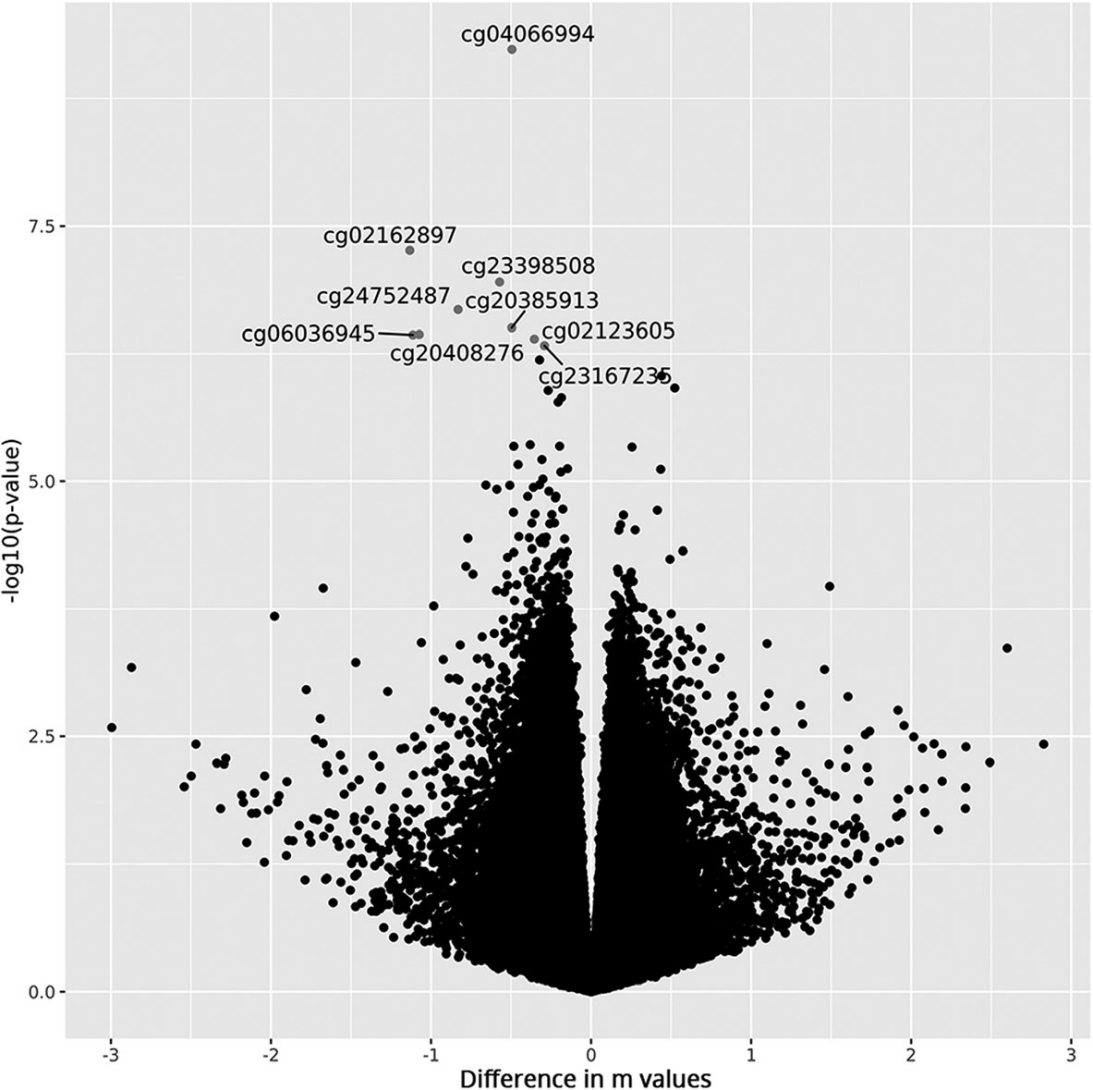
Volcano plot showing methylation differences of smokers compared to never smokers against -log10 of *p* values from the ANCOVA. Identifiers are given for the CpGs that were significantly associated with smoking after adjustment for multiple testing

The DNA of 18 healthy smokers and 21 healthy non-smokers of the same age was analyzed with the Infinium DNA MethylationEPIC BeadChip (Table 1). Next, 802,254 DNA methylation probes that passed the QC criteria were investigated for statistical significant differences in methylation levels between smokers and never smokers in an ANCOVA adjusting for age, sex and batch effects. A QQ plot revealed some global inflation of the test statistics as compared to the expected distribution, with an inflation factor of λ = 1.12 (Fig. 1a). After correction for multiple testing, we found nine CpGs to be significantly hypomethylated in smokers, with cg04066994 in the *AHRR* (aryl-hydrogen receptor repressor) gene showing the strongest association (*p* = 5.9 × 10^−10^, Δβ = − 0.08 in smokers; Fig. 1b, Table 2). The overall effect sizes of differentially methylated loci in smokers are illustrated in Fig. 2. Additionally, two significant CpGs mapped to the intronic region of *CYP1B1* and another one to an intergenic region 24 kb upstream of *CYP1B1*, three mapped to the long noncoding RNA *LINC00673* and one was located in an intergenic region ~ 6 kb upstream of *CYP1A2* (cytochrome P450 family 1 subfamily A member 2). Observed effect sizes were highest in *CYP1B1*, with Δβ = − 0.18 for cg02162897 (*p* = 5.5 × 10^−8^).

**Table 1:** Basic characteristics of the EWAS study population

	Smokers (*n* = 18)	Never smokers (*n* = 21)
Sex (males)	10 (55.6%)	8 (38.1%)
Age, years (mean)	38 ± 11	35 ± 11
SPY in smokers (mean)	12 ± 11	NA
SPY ≥ 10	9 (50%)	NA
SPY 2 > 10	9 (50%)	NA

**Table 2:** Change in DNA methylation β values for smokers compared to never smokers

CpG identifier	Chr	Position in bp (hg19^a^)	Gene symbol	Region	Δβ	*p* value^b^	*q* value
cg02162897	2	38,300,537	*CYP1B1* ^c^	Body	− 0.183	5.5 × 10^−8^	0.022
cg20408276	2	38,300,586	*CYP1B1* ^c^	Body	− 0.176	3.6 × 10^−7^	0.040
cg24752487	2	38,324,188	21 kb upstream of *CYP1B1*^c^	Intergenic; Mammalian H3K27me3-repression mark	− 0.138	2.1 × 10^−7^	0.040
cg06036945	5	394,869	*AHRR* ^c^	Body	− 0.125	3.7 × 10^−7^	0.040
cg04066994	5	394,891	*AHRR* ^c^	Body	− 0.083	5.9 × 10^−10^	4.7 × 10^−4^
cg20385913	15	75,054,510	6 kb downstream of *CYP1A2*	intergenic	− 0.063	3.1 × 10^−7^	0.040
cg02123605	17	70,497,550	*LINC00673* ^c^	Body	− 0.056	4.0 × 10^−7^	0.040
cg23167235	17	70,532,667	*LINC00673* ^c^	Body	− 0.018	4.7 × 10^−7^	0.042
cg23398508	17	70,536,199	*LINC00673* ^c^	Body	− 0.098	1.1 × 10^−7^	0.030

Shown are results for all CpGs significant after correction for multiple testing (*q* < 0.05). For comparison, we additionally included the results from the SPY analysis for significant CpGs

^a^NCBI build GRCh37

^b^*p* values are from the analysis of covariance (ANCOVA) with adjustment by age, sex and batch. *Chr* chromosome, *bp* basepairs, *SPY* smoke pack years, *kb* kilobasepairs

^c^Genetic region previously reported to be associated with smoking in buccals [22]

**Fig. 3:**
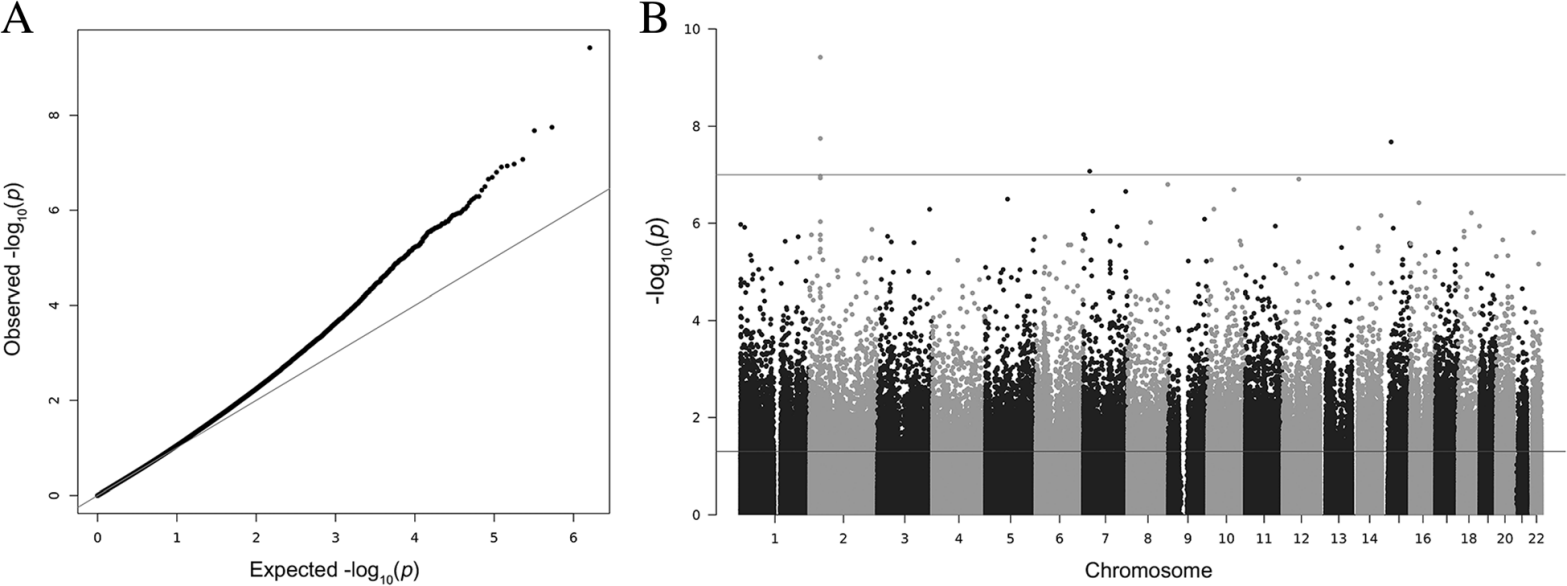
Manhattan and QQ pots for epigenome-wide associations with SPY in the masticatory mucosa. **a** The QQ plot showed some evidence for inflation of association signals (λ = 1.09). **b** Manhattan plot showing -log_10_ transformed *p* values from the ANCOVA plotted against the genomic location of the probes. The horizontal lines indicate the nominal significance threshold (*p* < 0.05) and the genome-wide significance threshold (*p* < 10^-7^)

**Fig. 4:**
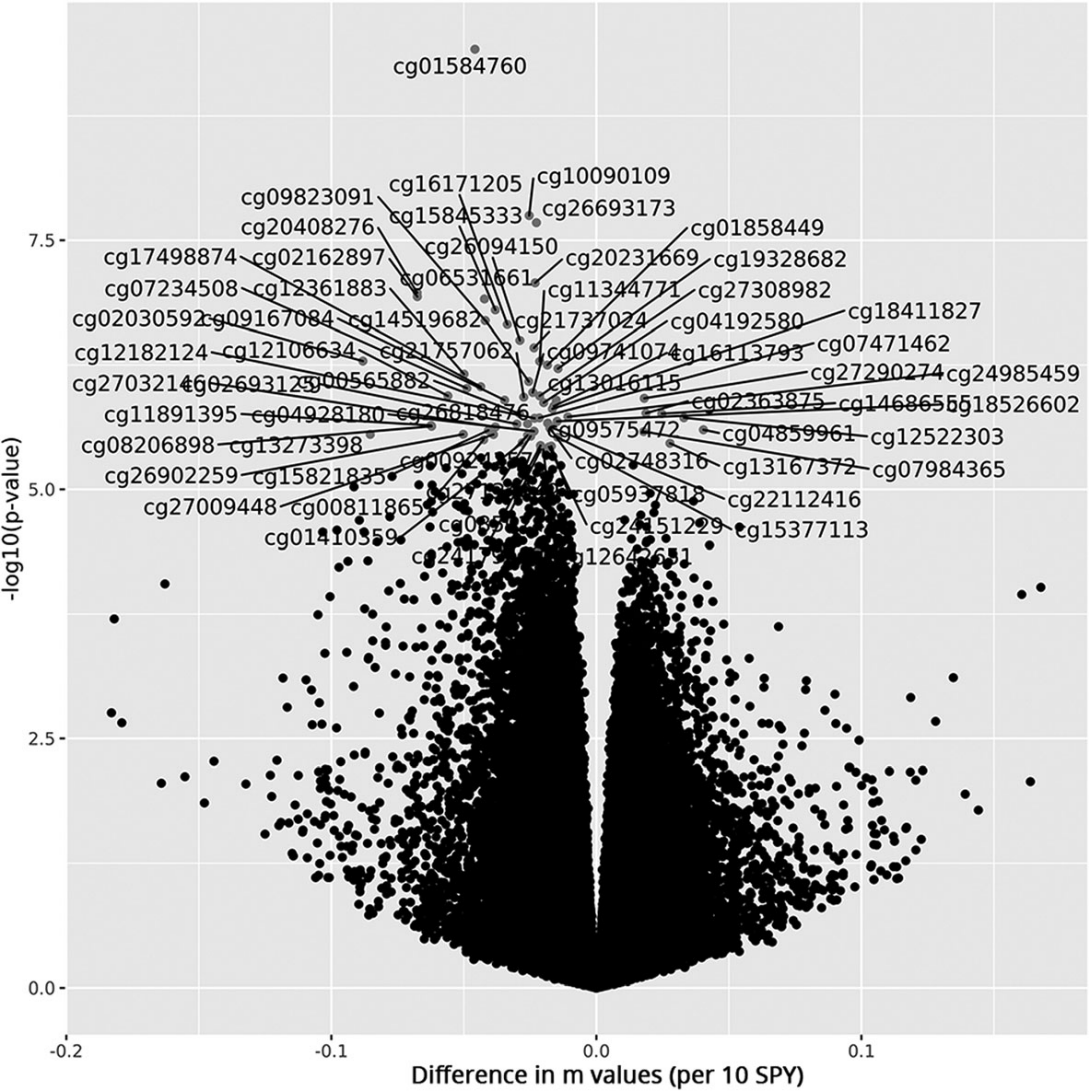
Volcano plot showing methylation differences per 10 SPY against -log10 of *p* values from the ANCOVA. Identifiers are given for the CpGs that were significantly associated with an increase in smoking after adjustment for multiple testing

Subsequently, the methylation levels were analysed for association with smoke pack-years (SPY), a measure for smoking intensity that includes information of the quantity of smoking. After correction for multiple testing, 54 CpGs showed significant hypomethylation and seven CpGs showed significant hypermethylation with increasing smoking levels, measured as SPY (*q* < 0.05; Figs. 3 and 4 and Table 3). The most significant differential methylation was observed in the 3′ untranslated region (3′UTR) of the gene *CYP1B1* (cytochrome P450 family 1 subfamily B member 1), with an average change in methylation of Δβ = − 0.046 per ten SPY at cg01584760 (*p* = 4.0 × 10^−10^; *q* = 3.2 × 10^−4^). Additionally, four other CpGs in the intronic region of *CYP1B1* showed significant hypomethylation with increasing SPY (e.g. cg20408276, Δβ = − 0.108 per ten SPY, *p* = 1.1 × 10^−7^). Another three significant probes (*q* < 0.05) flanked the long noncoding antisense RNA *CYP1B1-AS1*, which is partially overlapping with *CYP1B1* (e.g. cg10090109, Δβ = − 0.015 per ten SPY, *p* = 2.1 × 10^−8^). Among the other significant probes (*q* < 0.05), six showed similar or higher effect sizes compared to those observed at *CYP1B1*. These mapped to the genes *PIP4K2A* (phosphatidylinositol-5-phosphate 4-kinase type 2 alpha; cg02030592, Δβ = − 0.106 per ten SPY, *p* = 5.3 × 10^−7^), *DLX5* (distal-less homeobox 5; e.g. cg11891395, Δβ = − 0.097 per ten SPY, *p* = 2.5 × 10^−6^), *FABP4* (fatty acid binding protein 4; cg07234508, Δβ = − 0.073 per ten SPY, *p* = 1.1 × 10^− 6^), *FABP5P3* (fatty acid binding protein 5 pseudogene 3; cg16171205, Δβ = − 0.047 per ten SPY, *p* = 6.4 × 10^−7^) and *C10orf46* (cg27009448, Δβ = − 0.046 per ten SPY, *p* = 3.0 × 10^−6^). To account for our small sample size, which was very susceptible to false negative findings, we relaxed the significance threshold to *q* < 0.15 and compared the results of the SPY analysis to previously published results from a well-powered EWAS on smoking in buccal cells [22] and to a set of CpGs that was reported to be associated with smoking by multiple independent EWAS performed in blood [32]. Out of 352 CpGs that passed the lowered significance threshold in our data, 33 mapped to 18 loci that were associated in buccal cells, three of which were also reported in blood, among them *AHRR* (Table 4, Additional file 1). Moreover, two CpGs were associated with *q* < 0.15 that mapped to loci associated in blood, but not in buccal cells.

**Table 3:** Change in DNA methylation β values per ten smoking pack years for significant CpGs

CpG identifier	Chr	Position in bp (hg19^a^)	Gene symbol	Region	Δβ per 10 SPY	*p* value^b^	*q* value
cg09741074	1	1,310,496	*AURKAIP1*	5′UTR, 1st Exon	− 0.002	8.2 × 10^−7^	3.5 × 10^−2^
cg27290274	1	15,386,739	*KIAA1026* ^c^	Body	0.003	1.2 × 10^−6^	3.9 × 10^−2^
cg22112416	1	161,124,001	*UFC1*	Body	− 0.003	2.7 × 10^−6^	4.1 × 10^−2^
cg12522303	1	207,818,368	*CR1L*	TSS200	0.009	2.8 × 10^−6^	4.2 × 10^−2^
cg14686555	2	6,121,915	*LOC400940*	TSS200	0.005	2.1 × 10^−6^	4.1 × 10^−2^
cg08502446	2	36,891,478		Intergenic	− 0.017	3.5 × 10^−6^	4.6 × 10^−2^
cg01584760	2	38,296,474	*CYP1B1* ^c,d^	3′UTR	− 0.046	4.0 × 10^−10^	3.2 × 10^−4^
cg02162897	2	38,300,537	*CYP1B1* ^c,d^	Body	− 0.110	1.1 × 10^−7^	1.3 × 10^−2^
cg20408276	2	38,300,586	*CYP1B1* ^c,d^	Body	− 0.108	1.1 × 10^−7^	1.3 × 10^−2^
cg00565882	2	38,300,707	*CYP1B1* ^c,d^	Body	− 0.041	1.7 × 10^−6^	4.1 × 10^−2^
cg01410359	2	38,302,230	*CYP1B1* ^c,d^	Body	− 0.037	3.6 × 10^−6^	4.6 × 10^−2^
cg26818476	2	38,337,733	*CYP1B1*-*AS*	Upstream	− 0.021	2.3 × 10^−6^	4.1 × 10^−2^
cg10090109	2	38,338,120	*CYP1B1*-*AS*	Upstream	− 0.015	2.1 × 10^−8^	5.9 × 10^−3^
cg17498874	2	38,460,564	*CYP1B1*-*AS*	Downstream	− 0.062	1.1 × 10^−6^	3.9 × 10^−2^
cg04192580	2	223,726,348	*ACSL3*	5′UTR	− 0.008	1.3 × 10^−6^	4.0 × 10^−2^
cg24985459	3	37,035,090	*MLH1*	TSS200, 1st Exon	− 0.001	2.5 × 10^−6^	4.1 × 10^−2^
cg02748316	3	50,273,710	*GNAI2*	5′UTR	− 0.002	2.5 × 10^−6^	4.1 × 10^−2^
cg04859961	3	131,951,282	*CPNE4*; *tRNA*^*Cys*^	Body	0.024	2.6 × 10^−6^	4.1 × 10^−2^
cg11344771	3	188,108,786	*LPP*	5′UTR	− 0.020	6.5 × 10^−7^	3.1 × 10^−2^
cg26094150	5	79,378,871	*THBS4*	Body	− 0.003	2.9 × 10^−7^	2.2 × 10^−2^
cg24179483	5	170,814,656	*NPM1*	TSS200	− 0.002	3.1 × 10^−6^	4.3 × 10^−2^
cg09575472	5	174,045,109		Intergenic	− 0.032	2.0 × 10^−6^	4.1 × 10^−2^
cg07471462	6	33,168,624	*SLC39A7*	1stExon, 5′UTR	− 0.002	1.8 × 10^−6^	4.1 × 10^−2^
cg13273398	6	116,937,346	*RSPH4A*	TSS1500	− 0.018	2.7 × 10^−6^	4.1 × 10^−2^
cg26902259	6	126,102,246	*NCOA7*	TSS200	− 0.008	2.6 × 10^−6^	4.1 × 10^−2^
cg02363875	7	960,707	*ADAP1*	TSS200	0.003	1.7 × 10^−6^	4.1 × 10^−2^
cg18526602	7	6,487,739	*DAGLB*	TSS200	− 0.002	2.0 × 10^−6^	4.1 × 10^−2^
cg20231669	7	22,862,119	*TOMM7*	Body	− 0.005	9.2 × 10^−8^	1.3 × 10^−2^
cg01858449	7	33,551,455	*BBS9*	Body	− 0.020	6.2 × 10^−7^	3.1 × 10^−2^
cg27032146	7	96,652,115	*DLX5* ^c^	Body	− 0.062	2.4 × 10^−6^	4.1 × 10^−2^
cg11891395	7	96,652,153	*DLX5* ^c^	Body	− 0.097	2.5 × 10^−6^	4.1 × 10^−2^
cg21757062	7	121,376,168	329 bp from *7SK*	Intergenic	− 0.027	1.5 × 10^−6^	4.1 × 10^−2^
cg00924357	7	131,012,552	*MKLN1*	Body	− 0.001	2.6 × 10^−6^	4.1 × 10^−2^
cg16171205	7	152,135,221	*FABP5P3*	Body	− 0.047	3.0 × 10^−7^	2.2 × 10^−2^
cg15821835	8	67,686,767	*SGK3*	TSS1500	− 0.006	2.3 × 10^−6^	4.1 × 10^−2^
cg07234508	8	82,395,948	*FABP4*	TSS1500	− 0.073	1.1 × 10^−6^	3.9 × 10^−2^
cg15845333	8	144,417,025	*TOP1MT*	1stExon	− 0.003	1.6 × 10^−7^	1.6 × 10^−2^
cg14519682	9	130,213,309	*LRSAM1*	TSS1500	− 0.002	8.0 × 10^−7^	3.5 × 10^−2^
cg02030592	10	22,863,044	*PIP4K2A*	Body	− 0.106	5.3 × 10^−7^	3.1 × 10^−2^
cg09823091	10	94,644,717	*EXOC6*	Body	−0.033	1.8 × 10^−7^	1.6 × 10^−2^
cg08206898	10	116,581,183	*FAM160B1*	TSS1500	− 0.016	2.2 × 10^−6^	4.1 × 10^−2^
cg27009448	10	120,492,138	*C10orf46*	Body	− 0.046	3.0 × 10^−6^	4.3 × 10^−2^
cg12106634	11	108,092,400	*ATM*	TSS1500	− 0.006	1.2 × 10^−6^	3.9 × 10^−2^
cg06531661	12	57,522,120	*LRP1*	TSS200	−0.002	1.1 × 10^−7^	1.3 × 10^−2^
cg13016115	14	23,388,406	*RBM23*	TSS200	− 0.002	1.3 × 10^−6^	4.0 × 10^−2^
cg24151229	14	93,260,858	*GOLGA5*	5’UTR	− 0.002	3.6 × 10^−6^	4.6 × 10^−2^
cg15377113	14	93,799,418	*BTBD7* ^c^	TSS200	− 0.001	3.1 × 10^−6^	4.3 × 10^−2^
cg12361883	14	104,313,596	*LINC00637*	TSS1500	− 0.002	6.4 × 10^−7^	3.1 × 10^−2^
cg26693173	15	33,137,114	*FMN1*	Body	− 0.015	2.2 × 10^−8^	5.9 × 10^−3^
cg09167084	15	40,861,240	*RPUSD2*	TSS1500	− 0.008	1.1 × 10^−6^	3.9 × 10^−2^
cg04928180	15	99,791,525	*LRRC28*	TSS200	− 0.001	2.3 × 10^−6^	4.1 × 10^−2^
cg07984365	16	739,425	*WDR24*	1stExon	0.011	2.5 × 10^−6^	4.1 × 10^−2^
cg21737024	16	30,441,289	*DCTPP1*	1stExon	− 0.003	3.8 × 10^−7^	2.6 × 10^−2^
cg12642651	17	9,861,712	*GAS7*	Body	− 0.022	3.6 × 10^−6^	4.6 × 10^−2^
cg13167372	17	70,536,814	*LINC00673* ^c,d^	Gene body	0.027	2.2 × 10^−6^	4.1 × 10^−2^
cg12182124	18	21,451,563	*LAMA3* ^c^	TSS1500	− 0.040	2.2 × 10^−6^	4.1 × 10^−2^
cg16113793	18	21,451,607	*LAMA3* ^c^	TSS1500	− 0.028	1.8 × 10^−6^	4.1 × 10^−2^
cg19328682	18	47,807,700	*MBD1*	5’UTR	− 0.004	6.1 × 10^−7^	3.1 × 10^−2^
cg27308982	18	77,196,450	*NFATC1*	Body, 5’UTR	− 0.012	1.1 × 10^−6^	3.9 × 10^−2^
cg02693125	20	23,401,585	*NAPB*	Body	− 0.006	1.9 × 10^−6^	4.1 × 10^−2^
cg18411827	22	23,412,613	*GNAZ*	TSS200	− 0.003	1.4 × 10^−6^	4.0 × 10^−2^

Shown are results for all CpGs significant after correction for multiple testing (*q* < 0.05)

^a^NCBI build GRCh37

^b^*p* values are from the analysis of covariance (ANCOVA) with SPY as a continuous variable and adjustment by age, sex and batch. *Chr* chromosome, *bp* basepairs, *SPY* smoke pack years

^c^Genetic region previously reported to be associated with smoking in buccals [22]

^d^Shared association with the analysis of smoking status

**Table 4:** Change in DNA methylation β values per ten smoking pack years for validated CpGs from the literature

CpG identifier	Chr	Position in bp (hg19^a^)	Gene symbol	Δβ per 10 SPY	*p* value^b^	*q* value
Associations in buccals and blood						
cg04066994^c^	5	394,891	*AHRR*	− 0.04	8.2 × 10^−6^	0.07
cg14389122^d^	15	74,945,851	*EDC3*	− 0.05	1.3 × 10^−5^	0.08
cg17181940^c^	15	74,948,075	*EDC3*	− 0.05	5.6 × 10^−6^	0.06
Associations in buccals						
cg05223638	1	3,036,168	*PRDM16*	− 0.02	5.6 × 10^−5^	0.14
cg27290274	1	15,386,739	*KIAA1026*	0.00	1.2 × 10^−6^	0.04
cg01584760^c^	2	38,296,474	*CYP1B1*	− 0.05	3.8 × 10^−10^	3.2 × 10^−4^
cg02162897^d^	2	38,300,537	*CYP1B1*	− 0.11	1.2 × 10^−7^	0.01
cg20408276^d^	2	38,300,586	*CYP1B1*	− 0.11	1.1 × 10^−7^	0.01
cg00565882^d^	2	38,300,707	*CYP1B1*	− 0.04	1.7 × 10^−6^	0.04
cg01410359	2	38,302,230	*CYP1B1*	− 0.04	3.4 × 10^−6^	0.05
cg03890222	2	38,302,487	*CYP1B1*	− 0.02	6.2 × 10^−5^	0.15
cg21715189^d^	2	38,304,802	*CYP1B1*	− 0.06	3.2 × 10^−5^	0.11
cg06722870	2	236,840,187	*AGAP1*	0.00	6.0 × 10^−5^	0.14
cg04578109^c^	4	169,475,207	*PALLD*	− 0.01	1.5 × 10^−5^	0.08
cg21490342	6	31,852,301	*EHMT2*	0.01	6.5 × 10^−5^	0.15
cg23076086	6	112,194,849	*FYN*	− 0.01	2.1 × 10^−5^	0.09
cg18873386	7	96,651,915	*DLX5*	− 0.06	3.4 × 10^−5^	0.11
cg11367354	7	96,651,983	*DLX5*	− 0.05	1.1 × 10^−5^	0.07
cg27032146	7	96,652,115	*DLX5*	− 0.06	2.4 × 10^−6^	0.04
cg11500797	7	96,652,123	*DLX5*	− 0.07	7.1 × 10^−6^	0.06
cg11891395	7	96,652,153	*DLX5*	− 0.10	2.3 × 10^−6^	0.04
cg03532926	7	153,584,839	*DPP6*	0.01	1.3 × 10^−5^	0.08
cg06324554	7	157,495,841	*PTPRN2*	− 0.06	4.9 × 10^−5^	0.13
cg02583494^c^	9	97,640,188	*C9orf3*	− 0.03	4.2 × 10^−5^	0.12
cg22754866	10	99,771,121	*CRTAC1*	− 0.03	8.5 × 10^−6^	0.07
cg15377113	14	93,799,418	*BTBD7*	0.00	3.0 × 10^−6^	0.04
cg12344004	15	89,877,906	*POLG*	− 0.01	4.1 × 10^−5^	0.12
cg10099957	16	87,869,757	*SLC7A5*	− 0.06	9.6 × 10^−6^	0.07
cg09285525	16	87,869,786	*SLC7A5*	− 0.06	1.3 × 10^−5^	0.08
cg27555036	16	87,898,191	*SLC7A5*	− 0.03	4.5 × 10^−5^	0.13
cg13167372^d^	17	70,536,814	*LINC00673*	0.03	3.4 × 10^−6^	0.05
cg12182124	18	21,451,563	*LAMA3*	− 0.04	1.9 × 10^−6^	0.04
cg16113793	18	21,451,607	*LAMA3*	− 0.03	1.5 × 10^−6^	0.04
Associations in blood						
cg06566772^c^	10	63,661,030	*ARID5B*	0.00	1.7 × 10^−5^	0.09
cg16611234^d^	11	58,870,075	~ 4.5 kb from *FAM111B*	− 0.03	3.9 × 10^−5^	0.12

Shown are results for all CpGs that map to genes previously reported in a large EWAS on buccal swabs [22] or in a systematic review on EWAS on blood [32] and which were associated with SPY in our EWAS with *q* < 0.15

^a^NCBI build GRCh37

^b^*p* values are from the analysis of covariance (ANCOVA) with adjustment by age, sex and batch. *Chr* chromosome, *bp* basepairs, *kb* kilobasepairs, *SPY* smoke pack years

^c^Auxiliary probe on EPIC chip

^d^Same CpG reported in previous EWAS

### Estimation of different cell type fractions in the masticatory mucosa

**Fig. 5:**
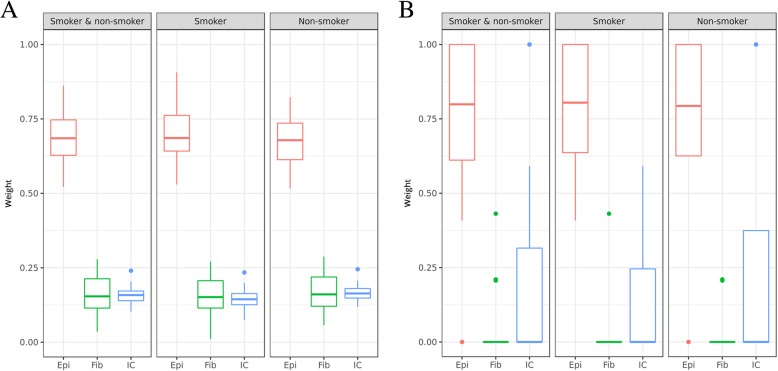
Cell type estimations in the masticatory mucosa inferred by EpiDISH. Shown are the boxplots of the average weight proportions of the major cell types epithelial cells (Epi), fibroblasts (Fib) and immune cells (IC) for EWAS (**a**) and OSCC samples (**b**). **a** EpiDISH estimations for the complete EWAS sample set (smokers and never smokers, *n* = 39) and for the subsets smokers (*n* = 18) and never smokers (*n* = 21). **b** EpiDISH estimations for the complete set of analysed OSCC samples (smokers and non-smokers, *n* = 16) and for the subsets smokers (*n* = 7) and non-smokers (*n* = 9)

The masticatory mucosa consists of a layer of keratinized epithelium, an underlying layer of connective tissue and invaded immune cells. We calculated the fraction of different cell types with the EpiDISH algorithm. The number of fibroblasts and immune cells in our samples was moderate (median 0.15 and 0.16, respectively) compared to epithelial cells (median 0.69; Fig. 5a, Additional file 2). The amount of epithelial cells in smokers and never smokers was highly similar (median 0.69 and 0.68, respectively). Similarly, we observed no differences in the estimated fractions of fibroblasts and immune cells, which seemed to be very slightly reduced in smokers (median of 0.15 and 0.14, respectively, compared to 0.16 for both cell types in never smokers).

### Differential gene expression between smokers and never smokers in the masticatory mucosa

We performed RNA-Seq of the same samples that were used in the EWAS to quantify the gene expression in the masticatory mucosa. Among the ten highest expressed genes in the solid oral mucosa, six were keratin genes (*KRT1*, *KRT6A*, *KRT10*, *KRT14*, *KRT16* and *KRT76* with median TPM values between 118,399 and 355,686; Additional file 3). The other genes of the top ten highest expressed genes were *S100A8* and *S100A9* of the S100 protein family, which has a prominent role in the regulation of inflammatory processes and immune response, *COX1* (cytochrome c oxidase subunit 1) and *EEF1A1* (elongation factor 1-alpha 1).

After adjustment for multiple testing according to Benjamini & Hochberg, 20 genes showed differential expression between smokers and never smokers (Table 5). The most significant association of differential expression and smoking status was found for *CYP1B1* (*p* = 2.2 × 10^−14^, *q* = 4.9 × 10^−10^, log2 fold change = 2.9). A KEGG pathway analysis showed a significant upregulation of two pathways, the Metabolism of Xenobiotics by Cytochrome P450 Pathway, (KEGG entry: rn00980; *p* = 3.7 × 10^−4^; *q* = 0.034; Table 6) and the Aminoacyl-tRNA biosynthesis pathway (KEGG entry: hsa00970; *p* = 1.9 × 10^−4^; *q* = 0.034). A Reactome pathway analysis showed 24 pathways to be significantly upregulated in smokers (*q* < 0.05). Out of these, 14 pathways belonged to the pathway classes of “cell cycle function” and/or “DNA replication” and three to the pathway class “metabolism” with respect to electron transport, most notably the top significant pathway involving the Citric Acid Cycle and Respiratory Electron Transport (Reactome identifier: R-HSA-1428517; *p* = 1.4 × 10^−5^; *q* = 0.009). In both approaches, no significantly downregulated pathways were found (Additional file 4).

**Table 5:** Differentially expressed transcripts in smokers compared to never smokers

Ensembl gene	Gene symbol	log2 fold change	*p* value	*q* value
ENSG00000138061	*CYP1B1* ^a^	2.9	2.2 × 10^−14^	4.9 × 10^−10^
ENSG00000181019	*NQO1*	1.2	1.2 × 10^−10^	1.3 × 10^−6^
ENSG00000176153	*GPX2*	1.0	1.0 × 10^−9^	7.7 × 10^−6^
ENSG00000108602	*ALDH3A1*	0.9	3.6 × 10^−9^	2.0 × 10^−5^
ENSG00000140465	*CYP1A1* ^a^	4.6	2.7 × 10^−8^	1.2 × 10^−4^
ENSG00000278270	*RPS9*	2.3	3.1 × 10^− 7^	0.0012
ENSG00000164236	*ANKRD33B*	− 0.7	6.2 × 10^−7^	0.0019
ENSG00000167165	*UGT1A6*	0.8	1.8 × 10^−6^	0.0042
ENSG00000171403	*KRT9*	1.3	1.9 × 10^−6^	0.0042
ENSG00000180574	*EIF2S3B*	− 3.2	1.7 × 10^−6^	0.0042
ENSG00000244122	*UGT1A7*	1.6	3.0 × 10^−6^	0.0060
ENSG00000197641	*SERPINB13*	0.6	3.7 × 10^−6^	0.0068
ENSG00000267023		5.2	7.4 × 10^−6^	0.0126
ENSG00000124588	*NQO2*	0.4	1.1 × 10^−5^	0.0179
ENSG00000117009	*KMO*	2.9	1.6 × 10^−5^	0.0226
ENSG00000118322	*ATP10B*	0.5	1.6 × 10^−5^	0.0226
ENSG00000284292		− 2.8	2.6 × 10^−5^	0.0335
ENSG00000135766	*EGLN1*	− 0.3	3.0 × 10^−5^	0.0368
ENSG00000119938	*PPP1R3C*	− 0.6	3.2 × 10^−5^	0.0375
ENSG00000135299	*ANKRD6*	− 0.4	4.5 × 10^−5^	0.0497

Differential expression analysis based on the RNA-Sequencing data. Log2 fold changes are given for the comparison smokers against never smokers

^a^Differentially methylated in our EWAS with *q* < 0.05

**Table 6:** Results from the pathway analyses for differentially expressed genes

Pathway	Identifier	Class	*p* value	*q* value
Reactome database				
The Citric Acid Cycle and Respiratory Electron Transport	R-HSA-1428517	Metabolism (electron transport)	1.4 × 10^−5^	0.009
tRNA Aminoacetylation	R-HSA-379724	Metabolism of proteins	1.1 × 10^−4^	0.019
Assembly of the pre-Replicative Complex	R-HSA-68867	DNA replication	1.3 × 10^−4^	0.019
M-G1-Transition	NA	Cell cycle	1.4 × 10^−4^	0.019
Cytosolic tRNA Aminoacylation	R-HSA-379716	Metabolism of proteins	1.7 × 10^−4^	0.019
HIV-Infection	R-HSA-162906	Disease	2.1 × 10^−4^	0.019
Host Interactions of HIV Factors	R-HSA-162909	Disease	2.1 × 10^−4^	0.019
Cell Cycle Checkpoints	R-HSA-69620	Cell Cycle	3.4 × 10^−4^	0.021
Destabilisation of mRNA by AUF1 (hnRNP D0)	R-HSA-450580	Metabolism of RNA	3.5 × 10^−4^	0.021
Respiratory electron transport, ATP synthesis by chemiosmotic coupling, and heat production by uncoupling proteins	R-HSA-163200	Metabolism (electron transport)	3.7 × 10^−4^	0.021
CDT1 association with the CDC6:ORC:origin complex	R-HSA-68827	DNA replication	3.7 × 10^−4^	0.021
Genes involved in APC/C:Cdc20 mediated degradation of mitotic proteins	R-HSA-176409	Cell cycle	5.4 × 10^−4^	0.026
Orc1 removal from chromatin	R-HSA-68949	Cell cycle, DNA replication	5.7 × 10^−4^	0.026
Regulation of mitotic cell cycle	R-HSA-453276	Cell cycle	6.0 × 10^−4^	0.026
Respiratory electron transport	R-HSA-611105	Metabolism (electron transport)	6.0 × 10^−4^	0.026
G1/S Transition	R-HSA-69206	Cell cycle	7.3 × 10^−4^	0.029
Regulation of ornithine decarboxylase (ODC)	R-HSA-350562	Metabolism	9.2 × 10^−4^	0.033
CDK-mediated phosphorylation and removal Cdc6	R-HSA-69017	Cell cycle, DNA replication	9.5 × 10^−4^	0.033
Autodegradation of the E3 ubiquitin ligase COP1	R-HSA-349425	Cell cycle	9.8 × 10^−4^	0.033
APC/C:Cdh1 mediated degradation of Cdc20 and other APC/C:Cdh1 targeted proteins in late mitosis/early G1	R-HSA-174178	Cell cycle	1.2 × 10^−3^	0.038
Metabolism of RNA	R-HSA-8953854	Metabolism of RNA	1.3 × 10^−3^	0.038
SCF-beta-TrCP mediated degradation of Emi1	R-HSA-174113	Cell cycle	1.4 × 10^−3^	0.039
Mitotic G1-G1/S phases	R-HSA-453279	Cell cycle	1.4 × 10^−3^	0.040
Mitotic M-M/G1 phases	R-HSA-453274	Cell cycle	1.5 × 10^−3^	0.040
KEGG database				
Aminoacyl tRNA Biosynthesis	hsa00970	Translation	1.9 × 10^−4^	0.034
Metabolism of Xenobiotics by Cytochrome P450	rn00980	Metabolism/xenobiotics biodegradation	3.7 × 10^−4^	0.034

We next investigated whether changes in methylation patterns have a measurable effect on gene expression in vivo. At a significance threshold of FDR = 0.05, *CYP1B1* was the only gene that showed correlation of hypomethylation with gene expression. Considering the limited statistical power of our sample that made it susceptible for false negative associations, we relaxed the significant thresholds and correlated all CpGs with *q* < 0.15 to those genes that were differentially expressed in smokers with a nominal *p* value of < 0.05. This analysis suggested differential expression of 15 genes with 25 CpGs at *q* < 0.15 (Additional file 5). To balance the resulting increased risk of type I errors, we subsequently included only those genes that mapped to at least two associated CpGs (*q* < 0.15) in our EWAS and had previously been reported to be associated with smoking in buccal swabs or blood [22, 32]. In total, four genes fulfilled these criteria: *CYP1B1*, *SLC7A5* (solute carrier family 7 member 5), *EDC3* (enhancer of mRNA decapping 3) and *LAMA3* (laminin subunit alpha 3), the latter of which showed opposite effect sizes for differential expression and differential methylation (Table 7). Because the differentially methylated lncRNA *CYP1B1*-*AS1* was not covered by our RNA-Seq reads, we analysed the expression by qRT-PCR in 69 individuals (*n* = 32 smokers, 37 never smokers). *CYP1B1*-*AS1* was stronger expressed in smokers when compared to never smokers with *p* = 0.0061.

**Table 7:** Correlation of differential methylation with differences in transcript levels

Gene	*p* value (DE)	*q* value (DE)	log2FC	Best CpG	Chr	Position in bp (hg19^a^)	Add. CpGs	*p* value (DM)^b^	*q* value (DM)	Δβ/10 SPY
*CYP1B1*	2.2 × 10^−14^	1.3 × 10^−10^	2.94	cg01584760	2	38,296,474	6	3.8 × 10^−10^	3.1 × 10^−4^	− 0.046
*SLC7A5*	1.1 × 10^−4^	6.5 × 10^−2^	0.62	cg10099957	16	87,869,757	2	9.6 × 10^−6^	7.1 × 10^−2^	− 0.060
*LAMA3* ^c^	0.025	0.64	− 0.20	cg16113793	18	21,451,607	1	1.5 × 10^−6^	4.0 × 10^−2^	− 0.027
*EDC3*	0.031	0.65	0.39	cg17181940	15	74,948,075	1	5.6 × 10^−6^	5.9 × 10^−2^	− 0.054

Genes nominally significant in the differential expression (DE) analysis that correspond to those CpGs that were associated with smoke pack years (SPY) with *q* < 0.15 in the differential methylation (DM) analysis and that lie within genetic regions previously reported to be differentially methylated in buccals [22]. *EDC3* was additionally reported to be differentially methylated in blood [32]

^a^NCBI build GRCh37

^b^*p* values are from the analysis of covariance (ANCOVA) with adjustment by age, sex and batch. *DE* differential expression, *Log2FC* log2fold change, *Chr* chromosome, *bp* basepairs, *Add. CpGs* additional CpGs significant with *q* < 0.15 at this locus, *DM* differential methylation, *SPY* smoke pack years

^c^Effect sizes for DE and DM are contradictory

### In vitro effects of cigarette smoke extract on the expression of hypomethylated genes in primary gingival fibroblasts

**Fig. 6:**
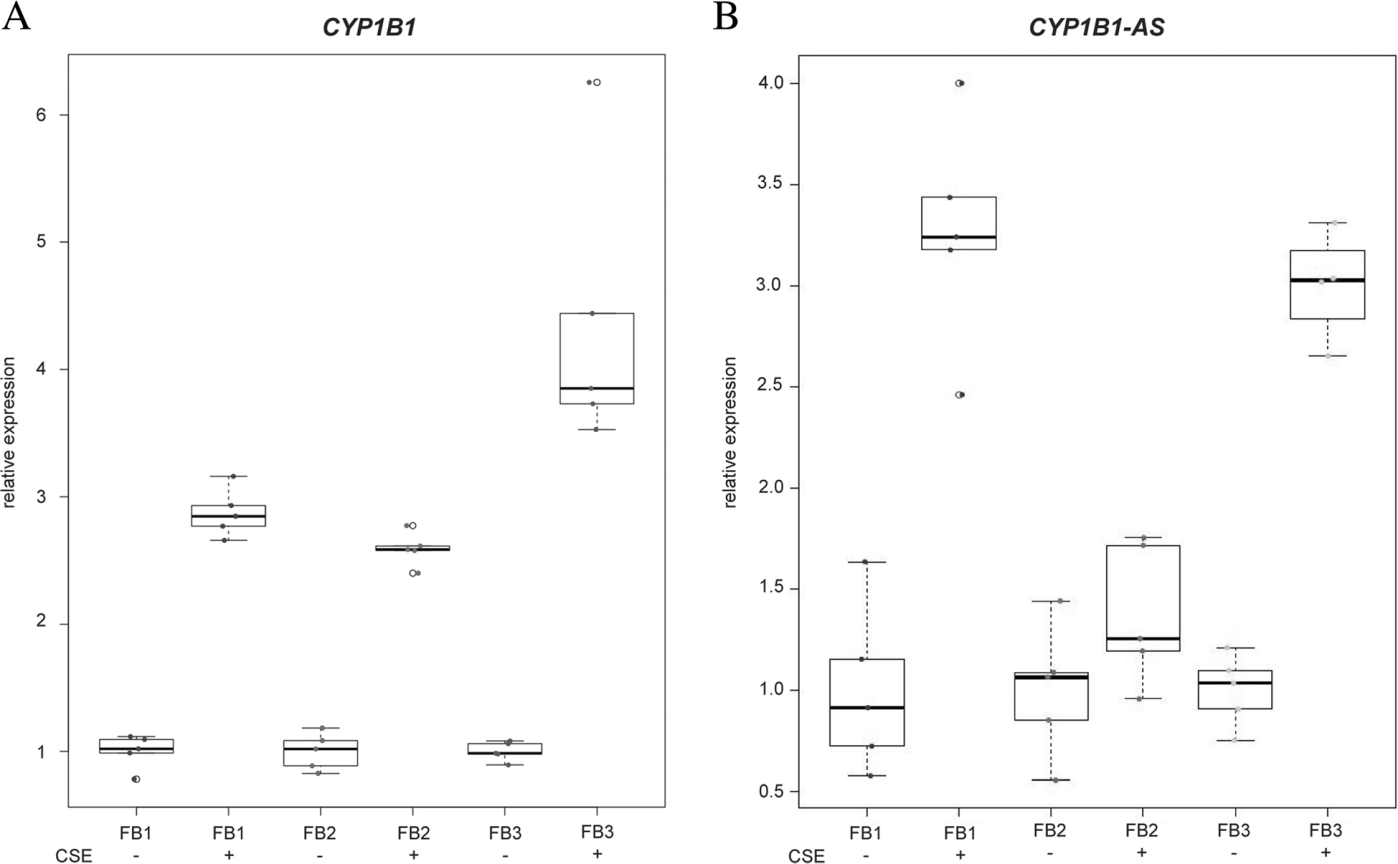
Gene expression of *CYP1B1* and *CYP1B1*-*AS* is significantly increased by cigarette smoke extract in vitro. Primary gingival fibroblast cell lines were cultured from three independent ex vivo biopsies (FB1–3) and treated for 6 h with cigarette smoke extract (CSE), labelled as “+”. Control cell lines without CSE treatment are labelled as “−”. qRT-PCR data were obtained from five treatments with CSE. Significant upregulation upon CSE treatment were observed for *CYP1B1* (p_adj_ = 0.019) and *CYP1B1-AS* (p_adj_ = 0.023). *P* values are Bonferroni-adjusted for two independent tests

*CYP1B1* had been shown to be induced in response to cigarette smoke exposure in the adenocarcinomic human alveolar basal epithelial cell line A549 from cancerous lung tissue [23]. To validate the regulatory effect of tobacco smoke on *CYP1B1* gene expression for gingival cells, we exposed gingival fibroblasts to cigarette smoke extract (CSE) in vitro. Exposure to CSE significantly increased the mRNA levels of *CYP1B1* and of the corresponding antisense RNA *CYP1B1*-*AS* with *p* = 0.019 and *p* = 0.023, respectively (Fig. 6).

### Association of smoking-induced changes in methylation levels in oral squamous cell carcinoma

To investigate a potential role of smoking-induced *CYP1B1* and *AHRR* hypomethylation in the development of oral cancer, we compared the methylation data from our healthy never smokers with the TCGA data for 16 oral squamous cell carcinoma (OSCC) biopsies that were specified as “gum” or “palate”, similar to the samples used in our EWAS. As the exact extraction site was not specified for OSCC samples, we first investigated cell type compositions using EpiDISH in this potentially heterogeneous tissue source. The cell composition varied extremely among OSCC samples, irrespective of smoking status (Fig. 5b, Additional file 2), with an overall higher amount of epithelial cells compared to our EWAS never smokers (median of 0.80 in OSCC compared to 0.69 in our samples). The OSCC data are based on the 450k array, and of a total of 202 CpGs at the genetic region of *AHRR* and *CYP1B1* that were analysed in our EPIC array dataset, 36% (*n* = 72) were not analysed with the 450k array. Yet, a total of 130 CpGs at *AHRR* (*n* = 98 CpGs) and *CYP1B1* (*n* = 32 CpGs) were shared between the OSCC samples and our data. In the OSCC data, at *CYP1B1*, 1 CpG was significantly hypomethylated and 16 CpG were significantly hypermethylated (corrected for multiple testing, *n* = 130 tests). At *AHRR*, 30 CpGs were significantly hypomethylated and 22 CpGs were significantly hypermethylated. The effect sizes in OSCC were high, with Δβ = − 0.48 for cg12202185 in *AHRR* (*p* = 2.8 × 10^−7^), and Δβ = 0.45 for cg09799983 in *CYP1B1* (*p* = 7.5 × 10^−12^), which both were not associated with smoking in our samples (Additional file 6). In our EWAS, five CpGs within *CYP1B1* showed significant hypermethylation in smokers, two of which were not analysed on the 450k chip for OSCC. The remaining three were significantly hypermethylated in OSCC, with the highest effect size at cg02162897 with Δβ = 0.197 (*p* = 4.7 × 10^−6^, Table 8). For *AHRR*, two CpGs were significantly associated in our EWAS but were not covered by the 450k chip used for the OSCC data. This is why we investigated the CpGs that flanked our associated CpGs in the EWAS, albeit these were not associated in our EWAS. In OSCC, one of these adjacent CpGs, cg04551776, showed a significant hypermethylation (Δβ = 0.15, *p* = 2.7 × 10^−5^).

**Table 8:** Associations of smoking and cancer with differential methylation of AHRR and CYP1B1

	CpG	Chr	Position in bp (hg19^a^)	Association with SPY (EWAS samples)			Association with smoking status (EWAS samples)			Association with OSCC		
				Δβ/10 SPY	*p*_nom_	*q*	Δβ	*p*_nom_	*q*	Δβ	*p*_nom_	*p*_adj_
*CYP1B1*	cg01584760^b^	2	38,296,474	− 0.046	3.8 × 10^−10^	3.1 × 10^−4^	− 0.045	1.0 × 10^−4^	0.853	Not analysed		
	cg02162897	2	38,300,537	− 0.11	1.2 × 10^−7^	0.014	− 0.183	5.5 × 10^−8^	0.022	0.197	4.7 × 10^−6^	6.1 × 10^−4^
	cg20408276	2	38,300,586	− 0.108	1.1 × 10^−7^	0.014	− 0.176	3.6 × 10^−7^	0.04	Not analysed		
	cg00565882	2	38,300,707	− 0.041	1.7 × 10^−6^	0.042	− 0.044	0.005	1	0.16	8.6 × 10^−7^	1.1 × 10^−4^
	cg01410359	2	38,302,230	− 0.037	3.4 × 10^−6^	0.047	− 0.032	0.023	1	0.177	8.8 × 10^−7^	1.1 × 10^−4^
*AHRR*	cg04551776	5	393,366	− 0.007	4.3 × 10^−1^	0.97	− 0.011	0.43	1	0.149	2.7 × 10^−5^	3.5 × 10^−3^
	cg06036945	5	394,869	− 0.067	1.0 × 10^−4^	0.18	− 0.125	3.7 × 10^−7^	0.04	Not analysed		
	cg04066994^c^	5	394,891	− 0.041	8.2 × 10^−6^	0.067	− 0.083	5.9 × 10^−10^	4.7 × 10^−4^	Not analysed		
	cg25648203	5	395,444	− 0.007	0.42	0.99	− 0.012	0.37	1	− 0.081	9.7 × 10^−3^	1

Shown are the results for all CpGs within *CYP1B1* and *AHRR* that were significantly associated with SPY levels or smoking status in our EWAS. We compared *AHRR* and *CYP1B1* methylation levels in 16 oral squamous cell carcinoma (OSCC) samples from TCGA to our healthy never smoker samples. Differential methylation analysis was performed using an ANCOVA adjusting for sex and unknown variation. The CpGs cg01584760, cg20408276, cg06036945 and cg04066994 were not covered by the 450k analysis of the OSCC samples. For *AHRR*, we added the results for cg04551776 and cg25648203, which flanked our EWAS-CpGs with a distance of 1503 bp and 553 bp, respectively, albeit these CpGs were not associated in our EWAS

^a^NCBI build GRCh37

^b^Top significant CpG in the differential methylation analysis with SPY

^c^Top significant CpG in the differential methylation analysis with smoking status. *Chr* chromosome, *bp* basepairs, *SPY* smoke pack years, *P*_adj_ Bonferroni-adjusted for 130 tests

## Discussion

In the current EWAS, we generated a reference dataset for combined differential gene methylation and expression patterns of the healthy masticatory mucosa of never smokers and smokers, accompanied by corresponding estimations of cell type fractions. We identified 63 significant differentially methylated CpGs after adjustment for multiple testing. Of these, the differential methylation at the gene *CYP1B1* had the highest effect size, with 11% hypomethylation per ten SPY. *CYP1B1* is a member of the cytochrome P450 gene family, and the gene product is involved in detoxification of pro-carcinogens in tobacco smoke [33]. *CYP1B1* was previously reported to be hypomethylated with epigenome-wide significance in EWAS of buccal swabs [22] and small airway epithelial cells of healthy smokers [27]. The significantly associated genes *PIP4K2A* and *DLX5* showed similar effect sizes as *CYP1B1*. Interestingly, the GWAS catalogue reports an association with variants within *PIP4K2A* for several cancer types. For *DLX5*, which was also found to be significantly associated with SPY in buccal cells [22], the GWAS catalogue reports variants associated with bone mineral densities and facial morphology. There is substantial evidence that smokers suffer from lower bone mineral densities [34, 35], possibly due to increased bone resorption [36]. Smoke-related toxins were described as inducing bone loss mediated by the AhR-pathway involving *CYP1A1*, *CYP1A2* and *CYP1B1* in mice [37]. Interestingly, in the analysis of smokers against never smokers as a categorical value, we found nine CpGs to be significantly differentially methylated, with the top two significant CpGs mapping to *AHRR* and *CYP1B1*, and another CpG 6 kb downstream of *CYP1A2*.

We consider the association of the non-coding RNA *LINC00673* as another interesting finding. This lncRNA was significantly hypomethylated in smokers in our study at three individual CpGs. This gene was shown to be upregulated in tongue squamous cell carcinoma [38].

Differential methylation of *AHRR*, *CYP1B1*, and *LINC00673* was shown before in buccal cells of smokers [22]. Increased expression of *CYP1B1* by cigarette smoke was shown in vivo in buccal cells after smoke stimulation [39], in bronchial airway epithelial cells from smokers [40] and in vitro in A549 lung adenocarcinoma cells [23]. *CYP1B1*-*AS1* was also shown to be upregulated in vitro by CSE exposure in A549 cells [23]. We could validate these findings in our differential expression analysis, showing a significant upregulation of *CYP1B1*, and additionally of *CYP1A1* in the masticatory mucosa of smokers and a CSE-induced upregulation of *CYP1B1* and *CYP1B1*-*AS* in vitro. Likewise, pathway analysis of differentially expressed genes revealed a significant upregulation of genes involved in the Metabolism of Xenobiotics by Cytochrome P450-KEGG pathway. Smoking is a widespread risk factor for periodontitis, a common inflammatory disease of the supporting tissues of the oral cavity, which is characterised by severe inflammation of the gingiva and irreversible bone degradation leading to tooth loss. With the data provided by this study, we add evidence to the significance of smoking on differential DNA methylation of cytochrome-P450-mediated AhR-pathway, a pathway that is involved in smoke-mediated bone degradation [37].

Apart from the upregulation of the Cytochrome P450-related pathway, Reactome pathway analysis of differentially expressed genes found 58% (14 out of 24) altered pathways relating to the classes DNA replication or cell cycle function, suggesting a connection of smoking and tumorigenesis.

Our data did not suggest a global correlation of the results from the differential methylation analysis with the differential expression analysis. However, we note that the majority of effect sizes in smoking EWAS are not large, with 12.5% hypomethylation in smokers for *AHRR* in our study. Therefore, the identification of subtle changes in expression caused by the methylation differences detected in EWAS requires very large sample sizes, even if assumed that the relationship between methylation and expression is linear. When accounting for other regulatory mechanisms that disturb this hypothesised linearity, the detection of a direct relationship between changes in methylation patterns and changes in expression patterns becomes even more difficult. Accordingly, EWAS that identified a large correlation of significant methylation with transcription differences are rare, and this despite the fact that hypomethylation is functionally associated with increased gene expression. However, we are aware of a large study that addressed the effects of smoking on methylation and transcription in 730 blood samples, which identified 254 CpGs showing significant correlation of differential methylation with expression values of nearby transcripts [13]. We presume that our non-finding of a global correlation is probably due to the lack of statistical power of the comparably small sample size. When filtering our expression data for genes with multiple associated CpGs and taking into account knowledge on previously published differentially methylated CpGs, in addition to *CYP1B1*, we identified two further genes, *SLC7A5* and *EDC3*, that showed consistent effect sizes for differential methylation and differential expression, and which were supported by smoking EWAS on buccal cells or blood. Variants within *EDC3* have been reported in a large meta-analysis on caffeine intake, together with *AHR*, *CYP1A1* and *CYP1A2* [41]. *EDC3* is located 25 kb upstream of *CYP1A1* and ~ 55 kb upstream of *CYP1A2*, which also harboured an associated CpG 6 kb upstream in our EWAS. Taken together, our results point towards an important role of differential methylation of several genes of the *CYP*-gene cluster on chromosome 15 to regulate the AhR-pathway in the masticatory mucosa of smokers.

In the comparison of *CYP1B1* methylation patterns of our never smokers data with the OSCC samples, all five significant CpGs were hypomethylated in our EWAS but in the OSCC data, *CYP1B1* showed global hypermethylation (16 out of 17 significant CpGs). For *AHRR*, findings are less clear because none of our CpG was analysed on the 450k chip used for the OSCC samples. Likewise, in 51 OSCC samples from various extraction sites (e.g. buccal mucosa, floor of mouth, lip, palate), significant downregulation of *CYP1B1* transcript levels was reported [42]. This contradicts the observation that *CYP1B1* was overexpressed in many cancers [43]. As suggested earlier [22, 23], our observations may indicate that demethylation and upregulation of *CYP1B1* in the oral mucosal tissues of smokers is not causal for the development of cancer, but in contrast, part of the normal cellular detoxification. In oral cancer, these pathways might be misregulated, which in turn would lead to impaired regulation of xenobiotic metabolism. However, these results contradict findings from other cancer types, where *CYP1B1* is overexpressed and serves as a tumour marker [44]. It needs to be emphasised that a major limitation of the OSCC dataset is that extraction sites were not specified. Cell compositions vary among different regions of the oral cavity [45], which is also suggested by the results of our cell type deconvolution analysis, and which could account for a stratification of results. As OSCC and other oral tissue samples from TCGA most likely have a heterogeneous cellular background originating from diverse tissue extraction sites, they might not be a perfect data source for a straightforward identification of differential methylation patterns. To investigate cell type-specific and disease-relevant methylation patterns in specific parts of the oral mucosa, further cell type deconvolution studies from different extraction sites and cell types of the oral mucosa can improve future analyses. The main limitation of our EWAS was the small sample size. Yet, our samples allowed the detection of the most significant tobacco smoke-related differentially methylated loci, which indicates the power of this homogeneously collected sample set.

## Conclusion

The current study adds evidence to previous EWAS that reported significant hypomethylation of *AHRR* and *CYP1B1* in buccal and airway epithelium of smokers. We could show increased expression of *CYP1B1* and *CYP1B1*-*AS* in the masticatory mucosa of smokers as compared to never smokers and validated upregulation of these genes by cigarette smoke in vivo and in vitro. We conclude that hypomethylation of *AHRR* and *CYP1B1* is an important regulatory mechanism of xenobiotic metabolism of the masticatory mucosa in response to tobacco smoke exposure.

## Methods

### Study sample

A total of 39 healthy smokers and never smokers, i.e. free of systemic disease, and all of European descent, were enrolled in this study, which was conducted in accordance with the principles of Good Clinical Practice and approved by the Independent Ethics Committee of each participating University Hospital. Written informed consent was obtained from all subjects according to the Declaration of Helsinki. All study participants completed a detailed questionnaire to provide general personal information (e.g. sex, age, geographical and family descent), information on the general and oral health and smoking habits (current, former, never smoker and amount of smoking in cigarette packs per day and duration of smoking in years). We calculated smoking pack year (SPY) values by multiplying the number of smoked cigarette packs per day by the number of years smoked. Fifty percent of the smokers had > 10 SPY, and 50% had between two and ten SPY. The mean age in smokers was 38 years and 35 years in never smokers. All individuals underwent periodontal examination prior to tissue extraction to exclude the presence of PD. Only individuals with a periodontal screening index (PSI) < 2 were included, indicating the absence of severe oral inflammation.

### Collection of ex-vivo tissue samples from the masticatory oral mucosa

Solid tissue samples from the oral masticatory mucosa were collected at a defined site from the hard palate adjacent to the 4th and 5th tooth by the use of a tissue puncher with 3 mm diameter and a depth of approximately 1 mm (for an illustration of the area of tissue sampling, see Additional file 7: Figure S1). We consider this site also representative for the gingival mucosa (i.e. often inflamed in periodontitis) because the gingival masticatory mucosa and the masticatory mucosa of the hard palate share the same histological cellular structures (Stratum basale, Str. spinosum, Str. granulosum, Str. corneum) and therefore the same cell types (Additional file 7: Figure S2). Likewise, indicating similar states of cellular differentiation, tissue collection at the specified sampling area is a common approach during periodontal surgery when free gingival grafts are excised and transplanted to other mucosal areas in the oral cavity. To stabilise DNA and RNA, the biopsies were stored in the AllProtect reagent (Qiagen, Hilden, Germany) immediately after punching, stored at 4 °C for 24 h, and subsequently transferred to − 20 °C.

### DNA and RNA extraction

The conserved tissue samples were manually broken up into small pieces with a scalpel and subsequently homogenised using the automated mixer mill MM 400 (Retsch, Haan, Germany) using frozen beads (3 mm, Retsch) for 90 s at 30 Hz. Subsequently, DNA and RNA was extracted simultaneously using the AllPrep DNA/RNA/miRNA Universal Kit (Qiagen, Hilden, Germany), ensuring the same cellular background of DNA and RNA for subsequent comparison of DNA-methylation and RNA-expression. Integrity of DNA and RNA was subsequently verified with a 2% agarose gel electrophoresis. Concentrations were measured using the Multiskan GO Microplate Spectrophotometer (Fisher Scientific, Hampton, USA). DNA and RNA samples were stored at − 80 °C.

### Bisulfite conversion and hybridisation to the Infinium MethylationEPIC BeadChips

Five hundred nanograms DNA per sample was bisulfite converted with the EZ-96 DNA Methylation Kit (Zymo Research, Irvine, USA) and hybridised to the Infinium DNA MethylationEPIC BeadChip (Illumina, San Diego, USA) on an iScan Microarray Scanner (Illumina) at the Institute for Clinical Molecular Biology, Christian-Albrechts-University Kiel, Germany.

### Differential DNA methylation analysis

For analysis and quality control, the R environment (Version 3.5.1) and the R package minfi (1.26.2) [46] were used, including the R package limma (Version 3.36.5) for the differential methylation analysis [47]. The Red/Green channel of the array were converted into one methylation signal without any normalisation. Using the function plotQC, we estimated sample-specific quality control (QC) and did not remove any sample. The QC criteria for probes were filtering of probes with median detection *p* values > 0.05, probes that lay within 5 basepairs (bp) of a single nucleotide polymorphism (SNP) with > 5% minor allele frequency, probes on the sex chromosomes and cross-reactive probes using the R package maxprobes (https://rdrr.io/github/markgene/maxprobes) according to Pidsley et al. [48] and McCartney et al. [49]. In total, 802,254  probes complied with the QC criteria and were included in the analysis. Methylation status was estimated according to the fluorescent intensity ratio, as any value between β = 0 (unmethylated) and 1 (completely methylated), and log2-transformed into *M* values, which are considered a more statistically valid estimator [50]. Corresponding effect sizes are, for a better biological understanding, given as Δβ, unless otherwise stated. After quality control, we performed a functional normalisation using the preprocessFunnorm function in minfi [51]. To identify CpGs correlating significantly with SPY, an analysis of covariance (ANCOVA) was performed for SPY as a continuous variable with adjustment by age as a continuous variable and sex as a categorical variable. We used SPY in this model to account for the cumulative risk exposure conferred by long-term smoking as suggested by Teschendorff et al. 2015 [22]. We additionally investigated differential methylation correlating with smoking status by performing an ANCOVA with the covariates age and sex. In both analyses, we additionally adjusted for technical variation, i.e. slide and position on slide, by using Combat and the R package BatchQc [52]. Correction for multiple testing was performed using the method by Benjamini & Hochberg [53]. CpGs were annotated to genes according to GRCh37/hg19 as provided in the MethylationEPIC BeadChip manifest.

### Cell type deconvolution of EWAS samples

To identify the presence of non-epithelial cells in our samples of oral mucosa, we used the EpiDISH algorithm (Version 3.9) [54], applying the centEpiFibIC.m.rda reference dataset on our beta-values, which includes centroids for epithelial cells, fibroblasts and immune cells.

### RNA-sequencing

To validate the effects of the observed methylation differences between smokers and never smokers on gene expression in the oral mucosa, we performed RNA-sequencing (RNA-Seq) of the same samples that were used in the EWAS. This ensured a homogeneous genetic and cellular background of the methylation and expression analysis. The total mRNA of 38 biopsy samples that passed QC for RNA-Seq (> 600 ng of total RNA, RIN > 7) was sequenced with a coverage of 16 million 50 bp single-end reads per sample. One RNA-sample was discarded due to low amounts of RNA. In brief, the library preparation was performed with 600 ng RNA using the TruSeq Stranded mRNA Kit (Illumina). RNA-Seq was performed on a HiSeq3000 (Illumina) at the Institute for Clinical Molecular Biology of the Christian-Albrechts-University Kiel, Germany.

### Differential expression analysis

RNA-seq samples were mapped to the transcriptome (Ensembl 96, GRCh38) using the transcript abundance quantification method Salmon [55]. Salmon estimates abundances without aligning reads. The transcript level estimates were then summarised for the gene level and imported into R using the package tximport [56]. Subsequently, the imported data was analysed for differential expression between smokers and never smokers using DESeq2 [57]. Genes with ≤ 2 counts in all samples combined were not considered in the analyses. To identify genes that show substantial evidence for a correlation of differential expression with differential methylation in smokers, we applied the following steps: (1) filtering of all differentially expressed transcripts with unadjusted *p* < 0.05; (2) filtering of transcripts with more than one CpG associated with q < 0.15 with SPY; (3) filtering of transcripts that were previously reported in a large EWAS on buccal swabs [22] or in a systematic review on EWAS on blood [32].

### Gene set enrichment analysis of differentially expressed genes

Gene sets of KEGG (*n* = 186) and Reactome (*n* = 674) were gathered from the Broad Institute Molecular Signatures Database (MSigDB [58, 59]). Together with the log2FoldChanges of the differential expression analysis, we performed an enrichment analysis using the R package gage [60]. Gene sets with *q* value < 0.05 were considered as being significantly enriched.

### Preparation of cigarette smoke extract and RNA extraction of cultured cells

Cigarette smoke extraction system and cigarette smoke extract (CSE) were prepared as described in [61]. CSE was prepared from Roth-Händle no filters cigarettes (Reemtsma, Hamburg, Germany). Smoke of three cigarettes was filtered using a 0.2-μM sterile filter and extracted into 10 mL cell culture medium. The CSE-containing medium was directly added to the cells (3 mL per well).

### CSE stimulation of primary gingival fibroblasts

For CSE stimulation, solid ex vivo biopsies of the masticatory oral mucosa of the hard palate were taken from three additional participants with 3 mm tissue punchers. Biopsies were dissected enzymatically to separate the epithelial cells from the fibroblasts by overnight incubation in 5 mg/mL dispase II (Sigma Aldrich) diluted in cell growth medium (DMEM, 1% Pen/Strep) at 4 °C. The primary gingival fibroblast cells (pGFs) were subsequently cultured in cell growth medium (DMEM, 1% Amphotericin B, 1% Pen/Strep, 1% non-essential amino acids). Prior to CSE stimulation, the pGFs (passage 3–4) were seeded in 6-well tissue culture plates (TPP Techno Plastic Products, Trasadingen, Switzerland) 24 h before stimulation (2.2 × 10^5^ cells per well). The CSE was freshly prepared at the day of stimulation. CSE induction was performed for 6 h in three biological replicates (each with five technical replicates) of pGFs, with aliquots of the same CSE. Three millilitre medium without CSE was added to the control cell plates (five technical replicates for each of the three donors). After 6 h of CSE incubation, the cells were washed twice with PBS. Cell disruption and total RNA extraction was carried out using the RNeasy Mini Kit (Qiagen) according to the manufacturer’s instructions.

### cDNA synthesis and qRT-PCR

To test differential expression in primary gingival fibroblasts upon CSE-stimulation, we performed quantitative reverse transcription polymerase chain reaction (qRT-PCR) for *CYP1B1* and *CYP1B1-AS*. qRT-PCR was performed with an amount of 500 ng of total RNA of CSE-stimulated cells using the High-Capacity cDNA Reverse Transcription Kit and oligo-(dT)-primers (Thermo Fisher Scientific, Waltham, USA) in accordance with the manufacturer’s guidelines, using oligo-(dT)- and dNTP-primers instead of random primers. Control PCR reactions contained water instead of cDNA. qRT-PCR experiments were performed using the CFX Connect System (Bio-Rad, Hercules, USA) in combination with SYBR Select Master Mix (Thermo Fisher Scientific) according to the manufacturer’s instructions. The gene expression levels were normalised to the mRNA expression of *GAPDH*, and relative expression was calculated using the mathematical model delta-delta ct (GraphPad Prism Software/R). Primers were manufactured (metabion GmbH, Planegg/Steinkirchen, Germany) with the following sequences: *CYP1B1* (forward: GAC GAC CCC GAG TTC CGT GA; reverse: AGC CAG GGC ATC ACG TCC AC) and *CYP1B1*-*AS* (forward: AGC CCT GAA AGA TGA ACA GTG GT; reverse: GGC ATG CCC ATT TCT TCC ACA). Because the lncRNA *CYP1B1*-*AS1* was not covered by mRNA-Sequencing, we analysed expression in our ex-vivo biopsies from the EWAS and 17 additional biopsies that were collected as described above with qRT-PCR. In total, *CYP1B1*-*AS*-expression of 32 smokers and 37 never smokers was analysed.

### Cell type deconvolution and differential methylation analysis of OSCC samples

To identify a putative role of genes that showed differential methylation in oral mucosa of smokers in cancer-development, we analysed data from The Cancer Genome Atlas (TCGA) [62]. We downloaded Illumina 450k methylation bead chip data from 16 samples of oral squamous cell carcinoma (OSCC) of keratinized oral mucosa (sites specified as “palate” or “gums”; i.e., Hard Palate, Upper Gum, Lower Gum and Gum NOS in the TCGA). To account for cell type-specific methylation, we performed the cell type deconvolution analyses on the 450k OSCC data as described above. We then performed an ANCOVA for all 130 CpGs that were annotated on the Illumina 450k array to the genetic regions of *CYP1B1* and *AHRR* with sex as a covariate and adjusting for technical variation using the R package sva [63] to compare OSCC samples to the healthy never smokers from the EWAS.

## Additional files

Additional file 1: CpGs from the literature associated with SPY in our data. Results are shown for the ANCOVA with SPY for all CpGs with q < 0.15 in our EWAS that map to genes harbouring a significant CpG in an EWAS on buccal swabs [22] or were reported as replicated in at least two independent EWAS on blood [32]. (XLSX 47 kb)

Additional file 2: Results of the EpiDISH cell type deconvolution. Estimates for the different cell type fractions are given each for the EWAS samples and the OSCC samples, each also additionally divided into the subsets smokers and non-smokers. (XLSX 17 kb)

Additional file 3: Median TPM values from RNA-Sequencing. Transcripts were ranked according to their median TPM value in the EWAS samples, with the highest expressed genes on top. (XLSX 1299 kb)

Additional file 4: Results from the KEGG and Reactome Pathway Analyses of Differentially Expressed Genes. (XLSX 76 kb)

Additional file 5: Correlation of associated CpGs with association of transcripts. Shown are all CpGs with q < 0.15 that map to genes that were differentially expressed in smokers with a nominal *p* value of < 0.05. (XLSX 14 kb)

Additional file 6: Results from the ANCOVA of OSCC samples in *AHRR* and *CYP1B1*. Listed are all CpGs within the genetic regions of *AHRR* and *CYP1B1* (*n* = 202) to indicate absence of CpGs in the 450 k analysis of OSCC. CpGs passing the adjusted significance threshold are marked in red. (XLSX 8961 kb)

Additional file 7: **Figure S1.** Area of tissue sampling. The tissue samples were collected from the hard palate directly adjacent to the 4th and 5th tooth by the use of a tissue puncher (3 mm diameter). **Figure S2.** Histological features of the masticatory mucosa of the gingiva and the hard palate. The mucosa of both sites is characterised by the four layered orthokeratinised stratified squamous epithelium, overlaying the lamina propria. The lamina propria contains closely packed bundles of collagen fibres enabling the mucosa to resist heavy loading. The cells in the upper keratin layer have lost their nuclei. Because the function and appearance of the cells and cell layers from both extraction sites are similar, it is the broad agreement of the periodontologists participating in this study that both sites are comparable and are likely to share similar methylation patterns under normal conditions. (DOCX 1137 kb)
