# Supplementary material for: A combined epigenome- and transcriptome-wide association study of the oral masticatory mucosa assigns CYP1B1 a central role for epithelial health in smokers
Source: Clin Epigenetics. 2019 Jul 22;11:105. doi: 10.1186/s13148-019-0697-y (PMC6647091; doi:10.1186/s13148-019-0697-y)
Supplement: Supplementary file 7 — Figure S1. Area of tissue sampling. The tissue samples were collected from the hard palate directly adjacent to the 4th and 5th tooth by the use of a tissue puncher (3 mm diameter). Figure S2. Histological features of the masticatory mucosa of the gingiva and the hard palate. The mucosa of both sites is characterised by the four layered orthokeratinised stratified squamous epithelium, overlaying the lamina propria. The lamina propria contains closely packed bundles of collagen fibres enabling the mucosa to resist heavy loading. The cells in the upper keratin layer have lost their nuclei. Because the function and appearance of the cells and cell layers from both extraction sites are similar, it is the broad agreement of the periodontologists participating in this study that both sites are comparable and are likely to share similar methylation patterns under normal conditions. (DOCX 1137 kb) [file 13148_2019_697_MOESM7_ESM.docx]

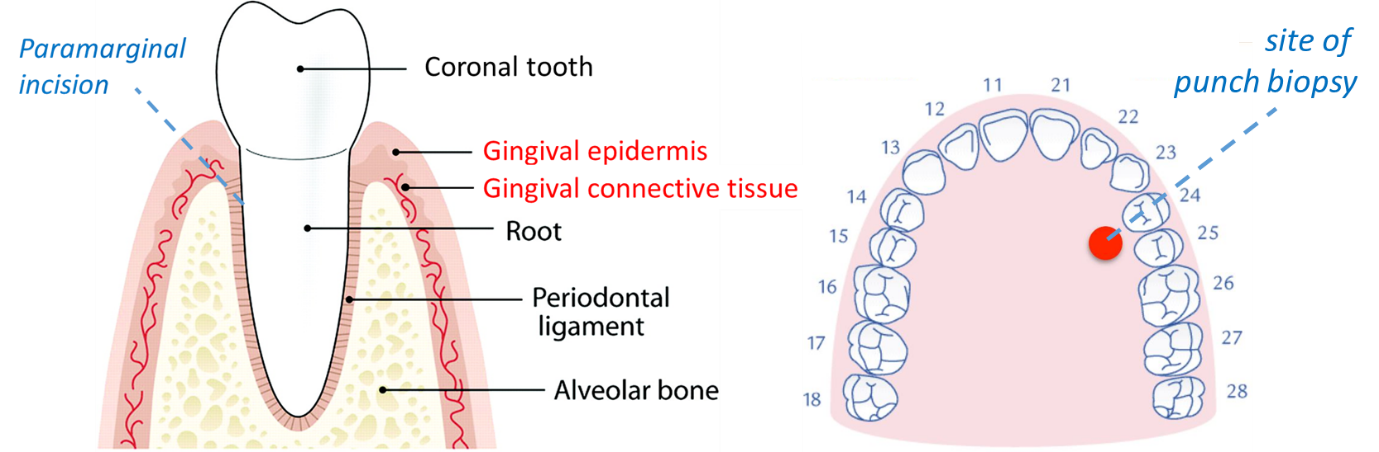
**Supplementary Figures**

**Figure S1. Area of tissue sampling**. The tissue samples were collected from the hard palate directly adjacent to the fourth and fifth tooth by the use of a tissue puncher (3 mm diameter).


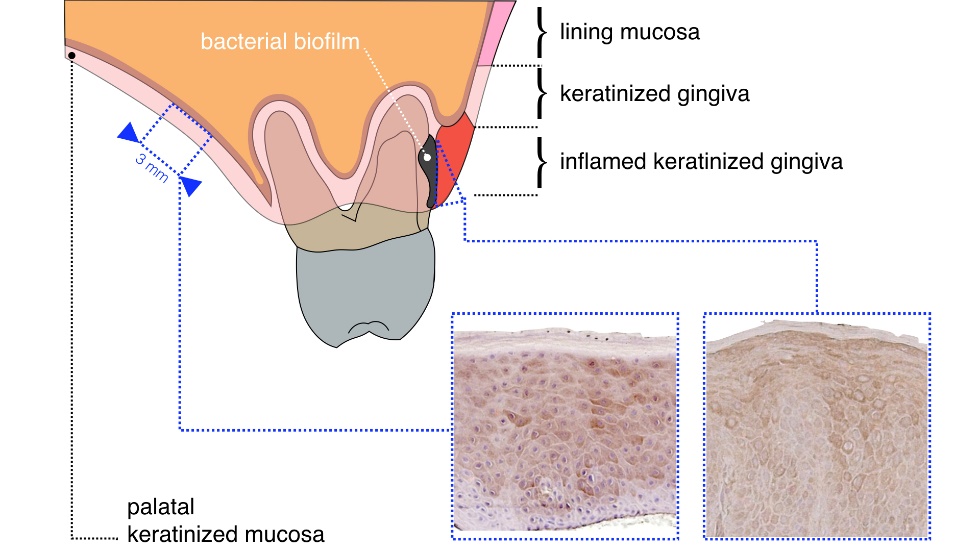


**Figure S2. Histological features of the masticatory mucosa of the gingiva and the hard palate.** The mucosa of both sites is characterised by the four layered orthokeratinised stratified squamous epithelium, overlaying the lamina propria. The lamina propria contains closely packed bundles of collagen fibers enabling the mucosa to resist heavy loading. The cells in the upper keratin layer have lost their nuclei. Because the function and appearance of the cells and cell layers from both extraction sites are similar, it is the broad agreement of the periodontologists participating in this study that both sites are comparable and are likely to share similar methylation patterns under normal conditions.
